# Molecular Mechanisms Underlying Stress Response and Resilience

**DOI:** 10.3390/ijms23169007

**Published:** 2022-08-12

**Authors:** Kazunori Kageyama, Takahiro Nemoto

**Affiliations:** 1 Department of Endocrinology and Metabolism, Hirosaki University Graduate School of Medicine, 5 Zaifu-cho, Hirosaki, Aomori 036-8562, Japan; 2 Department of Bioregulatory Science (Physiology), Nippon Medical School, Tokyo 113-8602, Japan

A variety of stressors induce various physiological responses by modulating sympathetic, neuroendocrine, and behavioral systems. Stress activates the hypothalamic–pituitary–adrenal (HPA) axis, whereas it suppresses the hypothalamic–pituitary–gonadal axis. The stress response also includes a physiological system for stress adaptation. The stressed state may be relieved or reduced by an action, medicine, or therapy. This Special Issue focuses on the stress response and resilience, which are related to their molecular mechanisms. In this Special Issue, two studies report the importance of elucidating stress mechanisms and stress-related translational research. Otubo et al. (2021) reported that, in Japanese macaque monkeys, both arginine vasopressin (AVP)/corticotropin-releasing factor (CRF) and the vesicular glutamate transporter 2/glutamate were co-localized in the magnocellular endings of the posterior pituitary and the parvocellular endings in the external layer of the median eminence [1]. Therefore, magnocellular and parvocellular AVP neurons are glutamatergic in primates. Theodoridi et al. (2021) generated a 11β-hydroxysteroid dehydrogenase type 2 (*Hsd11b2*) zebrafish mutant line to evaluate the involvement of this gene in stress-response regulation [2]. This new zebrafish model may represent an excellent tool for studying the response to different stressors and identifying cortisol-induced mechanisms during the stress response.

CRF in the hypothalamus is a crucial hormone to control the stress response. CRF in the hypothalamic paraventricular nucleus (PVN) regulates the HPA axis [3]. CRF receptor type 1 (CRF_1_ receptor) is mainly involved in stress responses, depression, anorexia, and seizures. The CRF_2_ receptor mediates “stress coping” responses, such as anxiolysis in the brain [3]. Orexin A upregulates CRF_1_ receptor expression, and CRF induces *Pomc* transcription [4]. Orexin A also suppresses bone morphogenetic protein (BMP)-4-induced intracellular signaling molecules [4]. Thus, orexin A has a stimulatory effect on *Pomc* expression by upregulating and downregulating CRF and BMP signaling, respectively. The orexin system is involved in the control of stress response and resilience. Pituitary BMPs are involved in the secretory control of follicle-stimulating hormone produced by gonadotroph cells. Soejima et al. (2021) elucidated the effects of *Clock* gene suppression and BMPs on the expression of luteinizing hormone (*Lh*) and *Clock* genes induced by gonadotropin-releasing hormone in gonadotroph cells [5]. Control of *Clock* gene expression by regulating the extracellular signal-regulated kinase pathway in the early phase and the BMP-Smad signal in the chronic phase could be a future strategy for modulating the LH surge [5].

Glucocorticoids are essential for stress coping, stress resilience, and homeostasis. The FK506-binding immunophilins FKBP4 and FKBP5 differentially regulate dynein interaction and nuclear translocation of the glucocorticoid receptor, resulting in the modulation of the glucocorticoid action [6]. FKBP4 contributes to the negative feedback of glucocorticoids, whereas FKBP5 reduces the efficiency of the glucocorticoid effect on proopiomelanocortin (*Pomc*) gene expression in pituitary corticotroph cells [6]. Low body weight at birth is a risk factor for future metabolic disorders, stress response abnormalities, and depression. Nemoto and Kakinuma (2021) observed impaired glucocorticoid responsiveness in low-body-weight rats due to malnutrition during the embryonic period in a pituitary-specific manner [7]. The authors also described that the expression of growth arrest-specific 5 (*Gas5*) lncRNA, a decoy receptor for the glucocorticoid receptor, increased only in the pituitary, and the expression of the *Fkbp5* against dexamethasone was induced in the liver, muscle, and adipose tissue. The dampened glucocorticoid response may be related to elevated *Gas5* lncRNA expression levels. Prenatal or postnatal methyl-modulator intervention can attenuate *Gas5* lncRNA expression in the pituitary.

Stress affects eating behavior. A complex network of appetite-related peptides controls appetite. Among various factors, ghrelin is a critical regulator of metabolism or energy homeostasis. Dexamethasone reportedly increased *Ghrelin* mRNA levels and its receptor mRNA and protein levels in hypothalamic cells [8]. Yamada (2021) demonstrated the relationship between the appetite-promoting peptide ghrelin and stress [9]. The orexigenic peptide, ghrelin, mediates stress-induced eating disorders by abnormal secretion and signal transduction. Disturbances in ghrelin signals are also influenced by aging and sex [9]. Sex differences regulate ghrelin responsiveness, and aging triggers a persistent stress response. 

Oxytocin is also stimulated by various stressful stimuli. Oxytocin is related to anxiety suppression and stress resilience [10]. The oxytocin system directly and indirectly exhibits adaptive changes dependent on internal and external conditions. Oxytocin in the hypothalamus has been shown to facilitate affiliative social behaviors and induce anxiolytic actions. Post-weaning stroking procedures reportedly induced affiliative responses via the activation of oxytocin neurons in the caudal PVN [11]. The oxytocin receptor also shows plastic changes during adaptation. Effective activation of the endogenous oxytocin system may strengthen the ability of the individual to adapt and facilitate resilience. Therefore, modulation of the oxytocin system may be valuable for preventing lifestyle-related or early life trauma-related diseases and health promotion. Al Jowf et al. (2021) provided an overview of various molecular biological, biochemical, and physiological alterations in post-traumatic stress disorder (PTSD), focusing on changes at the genomic and epigenomic levels [12]. PTSD is characterized by structural and functional brain changes. The underlying metabolic effects of conventional pharmacotherapies for PTSD are linked to epigenetic changes, which also occur throughout the disease progression.

Various factors contribute to stress resilience. All these factors have differing roles in stress resilience. The stressed state may be relieved or reduced via the HPA axis and other hormones by therapy, medicines, or natural stimulation. Successful therapy results in normal regulation of the HPA axis. To adequately treat stress-related diseases, molecular mechanisms underlying the stress response and resilience must be further explored.
